# Impact of sequential administration of detomidine, butorphanol, and midazolam on sedation, ataxia, stimulus response, and bispectral index in horses

**DOI:** 10.3389/fvets.2025.1691137

**Published:** 2025-10-23

**Authors:** Caitlin A. Thorn, Deborah V. Wilson, Sichao Wang, William A. Horne

**Affiliations:** ^1^Department of Small Animal Clinical Sciences, College of Veterinary Medicine, Michigan State University, East Lansing, MI, United States; ^2^Center for Statistical Training and Consulting, Michigan State University, East Lansing, MI, United States

**Keywords:** horse, sedation, midazolam, ataxia, bispectral index

## Abstract

**Background:**

Standing sedation is a safe and cost-effective alternative to general anesthesia in horses, but challenges include achieving adequate drug effect to block the stimulus response without inducing ataxia or recumbency. A benefit of midazolam has been reported in equine dental procedures. Seeking synergy, a combination of lower doses of several pharmacologic agents, including midazolam, may improve the quality of sedation while minimizing adverse effects. Bispectral index (BIS) correlates with sedation scores in human ICU patients, but the correlation between sedation scores and BIS has not been evaluated in horses.

**Objective:**

This study aimed to evaluate observational sedation scores and BIS in horses sequentially administered low-dose detomidine, butorphanol, and midazolam bolus and constant rate infusions (CRIs).

**Methods:**

Fifteen healthy horses received a standardized sedation protocol with sequential bolus doses and CRIs of detomidine, butorphanol, and midazolam. Sedation was assessed using a numerical rating scale that evaluated depth/stimulus response and postural instability/ataxia, and BIS was recorded at the same time points. Linear mixed-effect models assessed treatment effects; correlations between BIS and sedation scores were calculated within and between horses.

**Results:**

Sedation scores increased significantly with each drug added. The addition of midazolam increased sedation depth/reduced stimulus response (*p* = 0.01) and increased ataxia (*p* = 0.05). No horses became recumbent or displayed signs of excitement. Baseline BIS was 92 ± 4 (mean ± SD), decreased significantly after butorphanol administration (*p* < 0.001), and did not change significantly at any other evaluation point. Between-horse sedation scores were weakly correlated with BIS (*r* = −0.206; 95%CI: −0.664, 0.364; *p* = 0.478). Within-horse sedation scores were moderately correlated with BIS (*r* = −0.617; 95%CI: −0.756, −0.425; *p* < 0.001).

**Conclusion:**

In conclusion, the sequential addition of low-dose CRIs of butorphanol and midazolam to detomidine CRIs is associated with a stepwise increase in sedation and ataxia. Sedation score was not predicted by BIS. When sedating horses, low-dose midazolam may be added to improve sedation and reduce stimulus response, but the risk of pronounced ataxia should be considered.

## 1 Introduction

General anesthesia in horses is associated with a significant risk of mortality (1–3). This has led to the increasing use of local anesthesia and sedation to facilitate standing surgery for many invasive and painful procedures. Alpha-2 agonists are the cornerstone of equine sedation, but dose-dependent ataxia is a common side effect of this drug class (4, 5). Additionally, the use of alpha-2 agonists alone does not provide sufficient chemical restraint to facilitate invasive procedures such as oral examination or tooth removal in some horses (6–10).

Providing sufficient drug effect to blunt the response to surgical stimulus while minimizing postural instability and ataxia in standing horses presents a challenge for the practitioner. Wide ranges of detomidine doses have been reported, ranging from intravenous (IV) bolus doses of 0.0025 to 0.04 mg/kg and continuous rate infusions (CRIs) between 0.00625 and 0.04 mg/kg/h (3, 4, 8, 10–12). Seeking synergistic sedative and analgesic effects, alpha-2 agonists are usually combined with opioids such as butorphanol (4, 5, 10, 12, 13). Studied butorphanol doses ranged from IV bolus doses of 0.007 to 0.05 mg/kg and CRIs of 0.007 to 0.04 mg/kg/h (4, 6, 9, 12, 13).

Advantages of adding midazolam to dental procedures in horses have been reported (6, 9). Increased sedation depth, enhanced sedation quality, and occasional marked ataxia have been reported when a midazolam CRI (0.06 mg/kg/h) was administered following a bolus (0.02 mg/kg) in conjunction with romifidine CRIs or romifidine and butorphanol CRIs (6, 9). However, a single bolus of midazolam (0.02 mg/kg) added to detomidine and butorphanol did not produce a beneficial effect in one study (8).

Assessing sedation and predicting stimulus response in the sedated horse undergoing a surgical procedure can be difficult. Several subjective scoring systems have been developed to assess features such as facial expression, response to environment and to standardized stimuli, and observations of posture or ataxia (4, 14–16). The only objective assessment reported is head height above the ground (10, 17), but this variable is of limited utility if horses are restrained in stocks or a head rest is being utilized. Another objective measurement of sedation would be useful to assist practitioners in evaluating individual patients’ sedative depth.

Bispectral index (BIS) is a form of electroencephalographic (EEG) monitoring that combines EEG sub-parameters—bicoherence, power spectrum, and isoelectric activity—to calculate a single dimensionless score (0–100) that is correlated with the degree of cortical depression and therefore consciousness (18–20). In people, the following ranges have been established: 90–100 refers to awake state, 61–90 refers to moderate sedation to mild anxiolysis, <60 refers to deep sedation, 40–60 refers to general anesthesia, <40 refers to burst suppression, and 0 refers to no brain activity. Bispectral index also demonstrates moderate to strong correlation with clinical sedation scales in human adult ICU patients (21, 22). Several studies in horses report that BIS was not useful for assessing anesthetic depth (23–27). However, BIS correlation with clinical sedation scales in the standing horse has not yet been evaluated.

The objective of the present study was to use a published 7-point sedation score (14) to assess sedation depth, stimulus response, and postural instability or ataxia in horses following the sequential administration of low doses of detomidine, butorphanol, and midazolam. We also planned to monitor BIS at each of the time points when sedation was scored.

We hypothesized that (1) the addition of low-dose midazolam bolus and infusion to horses that are already sedated with low doses of detomidine and butorphanol would be associated with increased sedation and reduced response to stimuli without increasing postural instability; and (2) there would be a negative correlation between the sedation scores and recorded BIS values.

## 2 Materials and methods

### 2.1 Animals

This study was approved by the Michigan State University Institutional Animal Care and Use Committee (approval number: 201900345). It involved a longitudinal, within-subjects design consisting of 15 horses from the teaching herd at Michigan State University. These horses, aged 12 years (median, range = 3–30) and weighing 528 ± 47 kg (mean ± SD), included 1 gelding and 14 mares. The horses were of various breeds: American Standardbred (4), American Quarter Horse (3), Paint (2), Tennessee Walking Horse (2), Trakehner (1), and mixed breed (3). The horses were transported to the research barn in groups of two to three and allowed to acclimate for at least 12 hours. All horses were deemed healthy based on physical examination and routine hematology, including packed cell volume, total solids, and venous lactate measurements taken on the morning of the experiment. Food and water were not withheld.

### 2.2 Instrumentation

On the morning of the experiment, an intravenous catheter (14-gauge, 13 cm; MILACATH—extended use; MILA International Inc.; KY, USA) was aseptically placed in the left jugular vein of all horses. An extension set (High Flow Extension; MILA International Inc.; KY, USA) was attached for drug delivery, and both were secured using nylon suture (2–0 Ethilon; non-absorbable surgical suture USP; ETHICON INC.; NJ, USA). In preparation for arterial catheter placement, the skin over the transverse facial artery was clipped. At this time, in preparation for BIS sensor application (BIS Quatro 4 electrode sensor; Covidien, MA, USA), a strip of hair (3 cm x 15 cm) above the left eye was clipped from the mid-frontal bone to just above the lateral canthus. The skin was cleaned and defatted with 70% isopropyl alcohol.

At the start of the experiment, horses were walked into stocks, and intravenous crystalloid (lactated Ringer’s solution, Hospira Inc., IL, USA) administration was initiated at 2.5 mL/kg/h (28). After the placement of electrodes, heart rate and rhythm were monitored via electrocardiogram, and the BIS sensor was adhered to the skin with super glue and attached to the monitor (23).

Following the initiation of the detomidine infusion, the transverse facial artery was aseptically prepped and cannulated (20-gauge catheter, BD Insyte Autoguard; Becton Dickinson Infusion Therapy Systems Inc.; UT, USA). The catheter was secured in place with nylon suture as described above. Arterial blood gas analysis was performed (VetScan I-Stat1 Analyzer, i-Stat CG8 + Cartridge; Abaxis; CA, USA) immediately after catheter placement and at the end of the experiment. The catheter was attached to a calibrated transducer (Meritrans DTXPlus, DT-4812; Merit Medical, Singapore) and zeroed at the level of the right atrium for invasive blood pressure monitoring. Respiratory rate was monitored by observing chest excursions.

### 2.3 Experimental design

The study design was prospective, and each horse received the three studied drugs in the same order. A detomidine (Dormosedan; Zoetis; NJ, USA) bolus of 0.01 mg/kg was administered IV (T0) following baseline data recording. For infusion, detomidine was diluted in 0.9% NaCl to a final concentration of 0.05 mg/mL, and the CRI commenced at 0.01 mg/kg/h using a calibrated fluid pump (Baxter Flo-Gard 6201; Baxter Healthcare Corporation; IL, USA). The infusion was administered until study completion.

Thirty minutes following detomidine bolus administration, a butorphanol (Torbugesic; Zoetis; NJ, USA) bolus of 0.01 mg/kg was administered IV (T30) followed by CRI at 0.02 mg/kg/h using a calibrated syringe pump (Medfusion 3500; Smith Medical ASD Inc.; MN, USA). This continued with detomidine for the subsequent 60 minutes.

Thirty minutes following butorphanol bolus administration, a midazolam (midazolam; West Ward; NJ, USA) bolus of 0.01 mg/kg was administered IV (T60) and followed by CRI at 0.02 mg/kg/h using a separate syringe pump (Medfusion 3500; Smith Medical ASD Inc.; MN, USA). The three CRIs ran concurrently for 30 minutes.

### 2.4 Data collection

**Figure 1:**
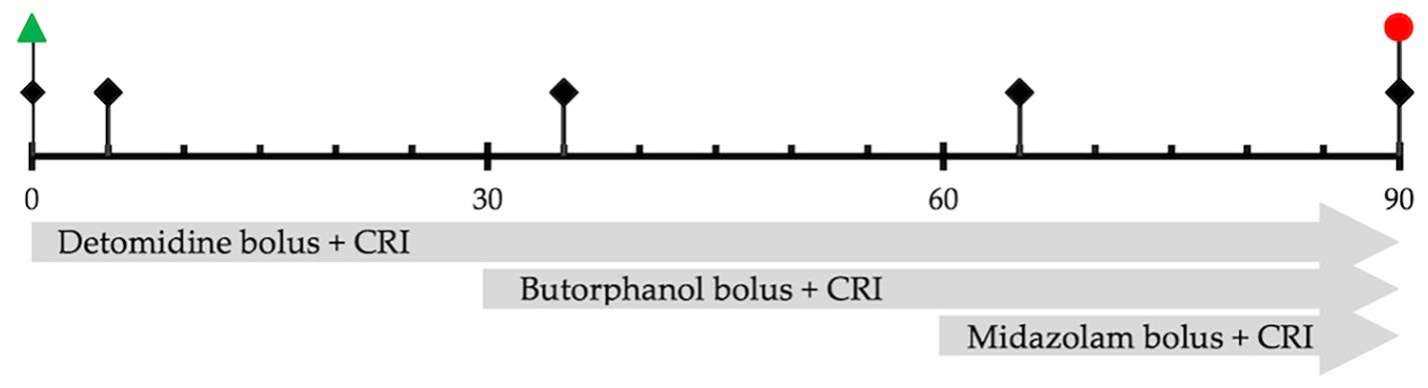
Study timeline represents the sequence of events and measurements over 90 min. 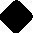 Physiologic measurements, sedation scores, and BIS values are recorded at the start of the experiment (before drug administration), 5 min after each drug administration, and at the end of all drug infusions. 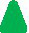 Start of drug infusion after recording baseline values. 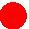 End all infusions.

The experimental timeline is depicted in Figure 1. After sensor placement, BIS was displayed continuously and reported as 5-minutes averages (23). The sensors were attached to the digital signal converter and monitoring equipment (Model A-2000; Aspect Medical Systems; MA, USA) and passed a system/sensor check for function and adequate contact. Impedance check, frequency filters, and artifact detection software were utilized prior to recording. Signal quality index, suppression rate, and electromyogram recordings contributed to the proprietary calculation and strength of the recorded BIS values (23, 24).

The physiologic values, 5-minute average BIS scores, and then sedation scores were recorded at the start of the experiment (Baseline, T0), 5 minutes after detomidine bolus and CRI (D, T5), 5 minutes following butorphanol bolus and CRI (DB, T35), 5 minutes following midazolam bolus and CRI (DBM, T65), and at the end of all CRIs (End, T90).

At the end of the 90-minute study period (T90), all drug administration was stopped, and a second sample of arterial blood gas was obtained. Horses were de-instrumented and monitored for an additional 10–20 minutes before safely walking back to the barn.

Sedation was scored using a modification of a widely reported numerical rating scale (Table 1), which evaluated first postural instability or ataxia and then depth of sedation and response to standardized stimuli (14). Assessment included observational and interactive components. Observational assessments were made first and included limb positioning, head ptosis, and facial characteristics. Interactive assessments included a whistle, abrupt “jumping jack” movement in front of the horse, attempted lingual exteriorization, and digital internal aural palpation, in that order. The scores from postural instability/ataxia (0–3) and depth of sedation/response to stimulus (0–3) were also combined for an overall score that ranged from 0 (no sedation) to 6 (maximal sedation). The individual assigning sedation scores (CT) was not blinded to treatments but was unaware of the BIS score.

**Table 1:** Sedation score description modified from Schauvliege et al. (14) with permission.

Postural instability/ataxia	Clinical signs
0	Standing square, bearing equal weight on all four legs. One hindlimb may be in a resting position.
1	Slight swaying or rocking. One limb may be in a resting position. Slightly lowered head height.
2	Clear swaying or leaning against the stocks (not bearing weight maximally on each of the four limbs). Limbs are not square beneath the body. Hindlimbs may be crossed.
3	Very pronounced leaning (not bearing weight on two or more limbs) and/or attempts to become recumbent. Hindlimbs are either crossed or extended caudally.
Sedation depth/stimulus response	
0	No sedation. Animal is alert with a normal posture and response to the environment/contact with the assessor. Normal objection to intervention. Ears are responsive to surroundings (moving).
1	Mild sedation. May or may not lean on head support, relaxed facial muscles. Reduced responses to background activity in the room and arm waving. Ears are partially responsive to surroundings and auditory stimuli. Light or no ptosis of the ears. Strong objection to pinnal palpation. Unable to keep the tongue exteriorized from the mouth.
2	Good sedation. Leans on the head support. No response to background activity in the room or arm waving. Blunted response to “jumping jack.” Pendulous lower lip. Ears are mildly responsive to auditory stimuli. Moderate ear ptosis. Eyelids are partially closed. Tongue remains exteriorized from the mouth. Blunted response to digital internal aural palpation.
3	Marked sedation. Leans strongly on head support. No response to all visual and auditory stimuli. Pronounced ear ptosis, minimal/no movement of the ears. Unresponsive to digital internal aural palpation. Eyelids are partially or fully closed. Eye may be rotated, with little to no movement of the eye.

© Association of Veterinary Anaesthetists and American College of Veterinary Anesthesia and Analgesia. Published by Elsevier Ltd. Modifications included the integration of stimulus responses to the sedation depth category and the elimination of the surgical quality category.

### 2.5 Statistical analysis

Five data points were used for all analyses: Baseline (T0), 5 minutes following bolus dose of each drug and during subsequent CRIs (T5, T35, T65), and at the end of all CRIs (T90). Linear mixed-effects models were used to evaluate all outcomes, with horses specified as a random intercept. Model assumptions were assessed using plots of conditional model residuals. The sedation scores were natural log-transformed to meet normality assumptions; a constant of 1 was added to allow transformation of zero values.

Categorical variables were summarized using frequencies. Continuous variables were reported as means ± standard deviations or medians with ranges, depending on distributional characteristics. The between-horse correlation between BIS and the combined sedation score was evaluated using Pearson’s product–moment correlation based on average values across time points for each horse. The within-horse correlation between BIS and the combined sedation score was assessed using the repeated measures correlation method (29). Herd size determined the sample size in this study. The minimum sample size was estimated based on several previously published studies reporting similar methodologies (6, 16, 30). Additional horses were studied to protect them from attrition. All analyses were performed using R (version 4.5.0; R Core Team, 2025) with the lme4, emmeans, rmcorr, and ggplot2 packages (31–35). Significance was reported with a *p*-value of ≤ 0.05.

## 3 Results

**Figure 2:**
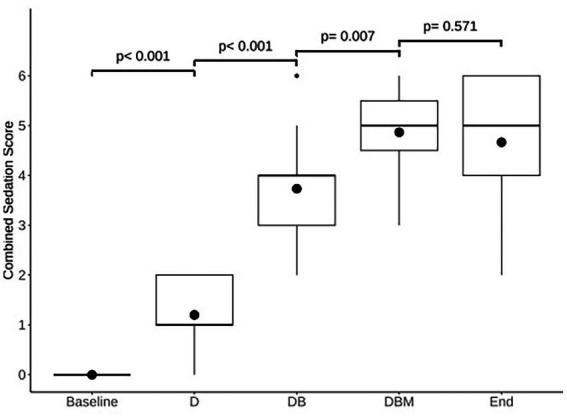
Comparison of modeled estimated means of combined sedation scores for each time point: the start of the experiment (Baseline), 5 min following detomidine (D), butorphanol (DB), and midazolam (DBM) administration, and at the end of all infusions (End). Box and whisker plot shows median (horizontal line), mean (circle), 25th–75th percentiles (box), 1.5 × IQR from 25th–75th percentiles 1.5 × IQR from 25th–75th percentiles (vertical lines), and outliers (dot). Numbers above connecting lines indicate *p*-values for each pairwise comparison.

Postural instability and ataxia increased significantly following the addition of each drug, from T0 to T5 (*p* < 0.001), T5 to T35 (*p* < 0.001), and T35 to T65 (*p* = 0.05), but not at completion of all CRIs (T65 to T90; *p* = 1.00). Sedation depth also increased, and stimulus response decreased significantly after the addition of each drug: T0 to T5 (*p* < 0.001), T5 to T35 (*p* < 0.001), and T35 to T65 (*p* = 0.011), but not at completion of the study, T65 to T90 (*p* = 0.374). Frequency distribution of the scores for postural instability/ataxia and sedation depth/stimulus response at each evaluation time is displayed in Table 2. Increases in the combined ataxia and sedation scores were also significant at each evaluation time (Figure 2).

**Table 2:** Frequency of distribution of sedation scores for horses (*n* = 15) at each data collection time point.

Score	Baseline		D		DB		DBM		End	
	Depth	Ataxia	Depth	Ataxia	Depth	Ataxia	Depth	Ataxia	Depth	Ataxia
0	15	15	7	5	0	0	0	0	0	0
1	0	0	8	10	3	5	0	2	2	2
2	0	0	0	0	10	8	6	7	5	7
3	0	0	0	0	2	2	9	6	8	6

Depth = sedation depth/stimulus response (0–3), ataxia = postural instability/ataxia (0–3) descriptive sedation scores (0–3) for depth and ataxia at the start of the experiment (Baseline), 5 minutes following detomidine (D), butorphanol (DB), and midazolam (DBM) administration, and at the end of all infusions (End). See Table 1 for definitions.

Following the administration of midazolam, some horses displayed signs of profound muscle relaxation; two horses became unresponsive to any stimuli, exhibited horizontal nystagmus, and lacked a menace response. None of the horses became recumbent or displayed signs of agitation or excitement at any point during the study. One horse was excluded from the BIS data collection due to poor sensor contact but was included in all other data collections. All 15 horses completed the experiment without complications and were able to walk back to their stalls within 20 minutes of discontinuing all drug administration.

Bispectral index scores varied widely between and within individuals (Table 3). Baseline (T0) BIS scores were 92 ± 4 (mean ± SD) and did not change significantly after detomidine administration (T5). Bispectral index decreased significantly following the administration of butorphanol (T35; *p* < 0.001). The BIS did not change significantly for the rest of the study. Agreement between BIS and sedation score between horses was poor (*r* = −0.206; 95%CI: −0.664, 0.364; *p* = 0.478). For (within) each horse, there was a negative correlation between BIS and sedation score that did reach significance (*r* = −0.617; 95%CI: −0.756, −0.425; *p* < 0.001).

**Table 3:** Mean and standard deviations of BIS values averaged over 5 min for horses (*n* = 14) at the start of the experiment (Baseline), 5 min following detomidine (D), butorphanol (DB), and midazolam (DBM) administration, and at the end of all infusions (End).

Variable	Baseline	D	DB	DBM	End
BIS	92 ± 4	93 ± 3	84 ± 10*	80 ± 10	82 ± 8

*Represents statistical significance between the previous time point.

Physiologic measurements remained within clinically acceptable ranges throughout the study (Table 4). There was a significant decrease in respiratory rate and heart rate, and a significant increase in mean arterial blood pressure from T0 to T5 (*p* < 0.001, *p* < 0.001, *p* = 0.012, respectively). The heart rate decreased significantly after T35 compared to T5 (*p* = 0.011). No other comparisons were found to be significant. Arterial blood gas measurements at the beginning and end of the 90 minutes were not clinically or statistically significantly different.

**Table 4:** Mean and standard deviations for respiratory rate (RR), heart rate (HR), and mean arterial blood pressure (MAP) for horses at the start of the experiment (Baseline), 5 min following detomidine (D), butorphanol (DB), and midazolam (DBM) administration, and at the end of all infusions (End).

Variable	Baseline	D	DB	DBM	End
RR	25 ± 9 n = 15	9 ± 6* n = 4	10 ± 4 n = 15	9 ± 5 n = 15	9 ± 5 n = 13
HR	36 ± 10 n = 15	24 ± 5* n = 14	28 ± 5* n = 15	30 ± 4 n = 15	29 ± 5 n = 13
MAP	82 ± 16 n = 2	96 ± 15* n = 4	91 ± 14 n = 15	95 ± 14 n = 14	95 ± 16 n = 14

See main text for statistical significance. *Represents statistical significance between the previous time point.

## 4 Discussion

The results of the present study did not align with our first hypothesis. The sequential addition of low-dose midazolam to detomidine and butorphanol CRIs was not only associated with a stepwise increase in the combined sedation score with reduced response to stimuli but also led to increased postural instability/ataxia. Selection of very low doses of both detomidine, butorphanol, and midazolam in the present study was anticipated to be associated with minimal side effects and a reduced likelihood of inducing ataxia, based on the review of several earlier studies of similar drug combinations (9–11, 17).

Benzodiazepines, such as midazolam and diazepam, are CNS depressants that enhance the inhibitory effects of the neurotransmitter GABA and produce muscle relaxation (36). This muscle relaxation is likely dose-dependent. Administration of midazolam alone (0.05 mg/kg IV) to study horses was reported to produce mild agitation and ataxia, with a median (range) distribution half-life of 24 minutes (6–42 min) (36). A second study administered midazolam at approximately half of that dose (0.02 mg/kg IV) to horses and reported no appreciable benefit or adverse side effects (8). We elected to study a bolus dose of midazolam, which was half of the latter dose, and a CRI that was one-third of the other doses published.

Advantages of midazolam for dental procedures in horses have been reported in previous studies (6, 9). In these studies, midazolam has been administered in doses at least double (0.02 mg/kg IV) the doses utilized in the present study, combined with an alpha-2 agonist and butorphanol, with the suppression of response to stimulus and increased sedation depth in some horses. An increase in ataxia was only reported in studies administering midazolam (0.06 mg/kg/h) infusions (6–9). We anticipated that the lowered dose of midazolam evaluated in the current study would be less likely to contribute significantly to any ataxia. We also anticipate that, when combined with an alpha-2 agonist and butorphanol, even doses lower than those studied will contribute to reduced response to stimulus and not impact postural stability.

The results of the present study do not support the use of BIS as a tool to facilitate assessment of the depth of sedation in the standing horse due to poor correlation of the BIS with sedation scores. Within each horse, BIS did tend to decrease as sedation increased, leading to a stronger correlation between BIS and sedation scores when assessed in each horse. Possible reasons for the lack of a dose-related decrease in BIS values in horses under either propofol, isoflurane, or sevoflurane anesthesia have been speculated and include the possibility of differing EEG patterns during inhalational anesthesia in the horse and the algorithm used to calculate BIS not being consistent in the horse (24, 26, 27). A variety of other reported factors that may contribute to the reduced quality and validity of the BIS include any adjacent external radiofrequencies, variable sensor contact, and muscle activity adjacent to the sensor (19). The inherent size, density, and shape variation of the equine skull compared to humans must also be considered (27).

The relatively small sample size, the fact that evaluators were not blinded, and the lack of a sham treatment could be considered limitations to the present study. In addition, the use of the widely reported Ghent Sedation Algorithm, which is a relatively low-range numerical rating system, may have limited our ability to detect small differences in effect between drugs (14, 16). The utilization of additional sedation scoring methods may also have improved the detection of differences between drugs. We used clinically utilized methods to control drug delivery and did not use target plasma concentrations, so it is possible that some decline or accumulation of plasma drug levels occurred during the CRIs. Distribution half-lives for both midazolam and detomidine are similar and are reported to be under 30 minutes (36–38). However, plasma concentration may not directly determine receptor effect. For consistency, we chose to evaluate the drug effect 5 minutes following bolus dosing to detect the peak drug effect as suggested by clinical experience.

In conclusion, sequential addition of low doses of butorphanol and midazolam to detomidine CRI is associated with a stepwise increase in sedation and increased postural instability. Bispectral index does not show benefit in assessing the level of sedation in horses. When sedating horses, low-dose midazolam may be added to improve sedation and reduce stimulus response, but the risk of pronounced ataxia should be considered.

## Data Availability

The original contributions presented in the study are included in the article/supplementary material, further inquiries can be directed to the corresponding author.
